# Tetanus After Trivial Trauma: A Case Series and Review of Pharamocotherapeutic Management

**DOI:** 10.7759/cureus.11089

**Published:** 2020-10-22

**Authors:** Nitin Gupta, Souvik Chaudhuri, Muralidhar Varma, Kavitha Saravu

**Affiliations:** 1 Infectious Diseases, Kasturba Medical College, Manipal Academy of Higher Education, Manipal, IND; 2 Critical Care Medicine, Kasturba Medical College, Manipal Academy of Higher Education, Manipal, IND

**Keywords:** clostridium tetani, diazepam, magnesium sulphate, atracurium

## Abstract

Tetanus is a vaccine-preventable disease that is still endemic in developing countries. We discuss two cases of tetanus in unvaccinated individuals. The first case was a young goat herder who was initially started on benzodiazepine and muscle relaxants. Magnesium sulphate was added later on due to inadequate control after which the patient improved significantly. The second case was an old farmer who was started on magnesium sulphate along with benzodiazepine from the outset. His clinical recovery was faster compared to the first case. We report these cases to discuss the pharmacological options in the management of generalized tetanus with particular reference to magnesium sulphate.

## Introduction

Tetanus is a vaccine-preventable neurological disorder that is caused by a spore-forming obligate anaerobe found in the soil [1]. Clinical tetanus is usually seen in unvaccinated individuals 3 to 21 days after a penetrating injury (leading to inoculation of spores of Clostridium tetani) [2]. The spore converts into the vegetative form in the damaged human tissue and produces the tetanospasmin toxin [1]. The tetanus toxin reaches the spinal cord via retrograde transport and inhibits presynaptic neurotransmission in the inhibitory motor neurons resulting in sustained muscular contractions. The patients with generalized tetanus usually present with trismus and progress to tonic muscular contractions and spasms. The symptoms progress in severity for up to two weeks and last for the total duration of 4-6 weeks [2]. The case-fatality rate of generalized tetanus is highest in areas with poor availability of resources such as mechanical ventilation [3]. Although the incidence of tetanus has drastically reduced in most parts of the world due to effective vaccination programmes, sporadic cases are still being reported [4]. We report two cases of generalized tetanus who presented to our hospital and was managed by our infectious disease team.

## Case presentation

Case 1

A 24-year-old male goat herder presented to our hospital with chief complaints of difficulty in mouth-opening for 12 days and spasms in all four limbs (upper limb followed by lower limb) for eight days. Eight days before the onset of symptoms, he reported an injury in the sole with a pointy wooden piece. He had no history of vaccination with tetanus toxoid. On examination, his vitals were stable, but he was found to have generalized rigidity (trismus, neck rigidity, abdominal guarding and rigidity of extremities). He was positive for spatula test. With a diagnosis of generalized tetanus, he was started on tetanus immunoglobulin, intravenous metronidazole and Tdap vaccine. For muscle relaxation, he was initially started on oral baclofen (four times daily) and intravenous diazepam (5mg four times daily). His diazepam was increased slowly over 48 hours to 10mg six times daily. He was sedated with this dose of diazepam, but his spasms continued. To protect the airways, he was tracheostomized as he could not be intubated due to inadequate mouth opening. He was shifted to midazolam (2mg per hour) and atracurium (0.6mg per hour). His midazolam was increased to 5mg per hour, and propofol was added after three days because of inadequate spasm control. There was some improvement in spasm control, but the course was complicated by the development of ventilator-associated pneumonia (Klebsiella pneumonia and Acinetobacter spp.). He was started on antibiotics according to susceptibility pattern (cefoperazone-sulbactam). He started to show improvement in ventilatory requirement after seven days of antibiotics. Due to inadequate control of spasms, despite twelve days of sedatives and neuromuscular blocking agents, intravenous magnesium sulphate was added at the rate of 2g per hour. It was titrated to keep the magnesium concentration between 5-6mg/dl. After magnesium sulphate was titrated, the spasms were controlled. Propofol and atracurium were discontinued while midazolam was de-escalated to diazepam tablet through Ryle’s tube for five days. His Creatine phosphokinase (CPK) levels reduced from 9711U/L at presentation to 354U/L. Magnesium was discontinued in another two days, but oral diazepam was continued. For seven days, his diazepam was stopped, he was weaned off the ventilator. He started to take orally and could walk with two-person support. At one month follow up, he reported that he was able to do all the routine activities on his own and was planning to go back to work.

Case 2

A 62-year-old male farmer, presented with difficulty in opening of mouth three days before the presentation at our hospital. He presented to the same hospital and team that managed Case 1 after six months of successful discharge of Case 1. This was followed by spasms in neck muscles, back muscles and all four limbs. Seven days before the onset of symptoms, he reported an injury in the dorsum of the left foot with a pointy wooden piece. He had no history of vaccination with tetanus toxoid. He had a history of unprovoked thrombus in the left lower limb three years back for which he was on oral anti-coagulants. On examination, he was tachypnoeic and was found to have generalized rigidity (trismus, neck rigidity, abdominal guarding and rigidity of extremities). He was positive for spatula test. With a diagnosis of generalized tetanus, he was given tetanus immunoglobulin and Tdap (combined tetanus, diphtheria and acellular pertussis) vaccine. He was tracheostomized and was started on intravenous metronidazole, oral baclofen (four times daily), intravenous diazepam (5 mg three times daily) and intravenous magnesium sulphate (2g per hour). Magnesium sulphate was titrated to keep the magnesium concentration between 4 and 5 mg/dl. After magnesium was titrated, the spasms were controlled over the course of six days. His CPK at admission was 1331U/L, which reduced to 256U/L in 14 days. His magnesium sulphate was stopped after 21 days. His hospital course was complicated with the development of hospital-acquired pneumonia (Pseudomonas aeruginosa) and Coronavirus disease-2019 (COVID-19). He was managed with intravenous antibiotics. He became ambulatory with active physiotherapy, and he was discharged eventually.

## Discussion

We report two cases of tetanus in two farmers after trivial trauma with sharp woody objects who presented to the infectious disease team of our tertiary care teaching hospital in southern India. Both the individuals were unvaccinated, highlighting the need for continued efforts in improving the vaccine coverage. The diagnosis of tetanus in both the cases was made clinically as is the norm. Other possible differentials like drug-related causes and poisoning were ruled out in both cases. Apart from the age and the time to presentation, most details in both cases were similar. However, the treatment approach that we took in both cases were different. Since the improvement of spasms in the first case was sub-optimal till the time magnesium sulphate was added, we initiated early magnesium sulphate in the second case. This could be one of the reasons for a faster recovery in the second case. The current evidence in the pharmacological management of tetanus is discussed herewith.

The treatment of tetanus is three-pronged and includes anti-toxin measures, control of spasms and airway management. Penicillin or metronidazole for 7-10 days along with wound debridement is required to eradicate Clostridium tetani to stop further production of toxins. In a non-randomized controlled trial published in 1985, metronidazole was shown to have improved outcomes when compared to penicillin [5]. However, in a subsequent randomized clinical trial from India, no difference in clinical outcomes was observed between penicillin and metronidazole [6]. To neutralize the unbound toxins, intramuscular tetanus immunoglobulins is recommended at a dose of 500 units. Although a randomized controlled trial from Brazil showed better clinical outcomes with intrathecal immunoglobulins, the intramuscular route is preferred in routine practice due to the ease of administration [7]. Since clinical infection is not enough to induce an adequate immunological response, vaccination with tetanus toxoid is recommended in all patients with clinical tetanus.

The patient should be placed in a dark and quiet room to avoid physical stimuli (light, sound) that can provoke or exaggerate spasms. The various agents that can be used for control of spasms and autonomic instability are summarized in (Table 1) [8,9]. Benzodiazepines (diazepam, midazolam) are used for their sedative effect along with their ability to control spasms. General anaesthetic agents such as propofol can also be used. Often the sedatives are not enough to attain adequate relaxation and neuromuscular blocking agents such as pancuronium, vecuronium and atracurium are used additionally to control the spasms. Baclofen is a postsynaptic GABA receptor stimulant that can also be used. Retrospective studies have shown a good efficacy of intrathecal baclofen, but this route is associated with a risk of nosocomial meningitis [10]. There is scant literature on the use of oral baclofen in tetanus. 

**Table 1:** Drugs used for spasm control in patients with tetanus Abbreviations: IV- intravenous, GABA- Gamma-aminobutyric acid, NM- neuromuscular blocker, h-hour, min- minute, f/b- followed by

Drugs	Mechanism of action	Role in tetanus	T_1/2_	Dose	Adverse events	Advantages/disadvantages
Diazepam (IV)	Benzodiazepines-Binds to receptors on postsynaptic GABA and enhances its inhibitory effect	Relief of muscle spasms	87h	10-30 mg q1-4h (as high as 100 mg/h)	Drowsiness Hypotension	Cumulation of metabolites- Metabolic acidosis
Midazolam (IV)			3h	0.02-0.1 mg/kg/h		Less cumulation of metabolites
Magnesium sulphate	Presynaptic NM blocker blocks catecholamine release and reduces responsiveness to receptor	Relief of muscle spasm, Reduces autonomic instability		40 mg/kg loading followed by 2g/h	Hypotension Cardiotoxicity	Needs close monitoring
Baclofen (oral)	Stimulates postsynaptic GABA receptors	Relief of muscle spasms	3-4.5h	5-10 mg TDS (to be titrated up to 60 mg/day)	Respiratory depression Cardiovascular instability	Limited evidence
Intrathecal baclofen			1-5h	500-2000 μg/day		Chance of CNS infections
Vecuronium	Neuromuscular blocking agents -Blocks acetylcholine from binding receptors	Relief of muscle spasms	70m	0.1 mg/kg bolus f/b 0.1 mcg/kg/min	Prolonged use- Critical illness neuropathy/ myopathy	No autonomic instability but shorter-acting
Pancuronium			80-160m	0.1 mg/kg bolus f/b 1 mcg/kg/min		Longer-acting but autonomic instability
Atracurium			2-20m	0.5 mg/kg bolus f/b 5-10 mcg/kg/min		
Propofol	Agonism of GABA receptors	Relief of muscle spasms	40m-4h	5 mcg/kg/min- increase the dose to maintain at 5-50 mcg/kg/min	Hypotension Apnoea, Involuntary movements	Rapid recovery after stopping
Clonidine	Central sympatholytic -Partial agonist α2 receptor	Reduces autonomic instability	8-12h	100 μg q8-12h	Bradycardia Hypotension Respiratory depression	
Dexmedetomidine	Central sympatholytic -Full agonist α2 receptor		2-2.5h	1 μg/kg bolus f/b 0.5 μg/kg/h	Bradycardia Hypotension	
Morphine	μ, kappa-opioid receptor agonist		2-3h	0.1mg/kg bolus f/b 1-2mg/h	Respiratory depression Bradycardia, hypotension	Causes Cardiovascular stability
Fentanyl	μ-opioid receptor agonist		219m	2 μg/kg bolus f/b 1 μg/kg/h infusion	Respiratory depression	

Magnesium sulphate is a presynaptic neuromuscular blocker that reduces autonomic instability by reducing the release of catecholamine and responsiveness of receptors to catecholamines8. It also decreases the requirement of other drugs to control spasms. In a well-controlled randomized placebo-controlled study from Vietnam, magnesium sulphate did not decrease the requirement of mechanical ventilation. Still, it decreased the requirement of other drugs to control the spasms [11]. In an unblinded small trial from Nigeria, magnesium sulphate was superior in decreasing the frequency of spasms when compared to diazepam. However, it was not superior in decreasing mortality [12]. Similar results were reported from another unblinded study from Pakistan [13]. The airway is an essential aspect of management as generalized spasms are associated with respiratory failure. Besides, the high dose of sedatives and paralytic agents required to control the spasms require preemptive airway protection. Therefore, early intubation or tracheostomy is beneficial in the overall management of patients with generalized tetanus [3].

Generalized tetanus is associated with a high fatality rate in developing countries with reports of it ranging from 37 to 58%. Studies from Brazil, Turkey and India have consistently reported a shorter incubation period, higher age and early onset of spasms/severe symptoms as predictors for mortality [14-16]. Involvement of respiratory system necessitating prolonged ventilation is a predictor of mortality in resource-limited settings. This is because of either the lack of facilities or the cost associated with prolonged ventilation. The development of ventilator-associated pneumonia further complicates this with multi-drug gram-negative resistant organisms due to inadequate infection control practice. In the current scenario, with the COVID-19 pandemic, the management is becoming all the more challenging with patients in intensive care units contacting COVID-19. It is pertinent to institute effective infection control procedures to prevent these infections. In both our cases, there were multiple risk factors for mortality, but effective pharmacological management was the key to a good outcome in both cases. 

## Conclusions

In summary, the first case was a young unvaccinated male who presented with features of generalized tetanus after a history of trivial trauma. He was initially managed with benzodiazepines and muscle relaxants, but adequate spasm control was achieved only after initiation of magnesium sulphate. The second case was an old unvaccinated male who also presented with similar features. The second case was started upfront with magnesium sulphate and showed prompt control of spasms. In conclusion, magnesium sulphate is a key pharmacological intervention that can be used to control muscle spasms in patients with tetanus. We report this case series to discuss the challenges faced in the management of tetanus and the role of different pharmacological interventions in the successful management of such cases. 
